# LI Detector: a framework for sensitive colony-based screens regardless of the distribution of fitness effects

**DOI:** 10.1093/g3journal/jkaa068

**Published:** 2021-03-06

**Authors:** Saurin Bipin Parikh, Nelson Castilho Coelho, Anne-Ruxandra Carvunis

**Affiliations:** Department of Computational and Systems Biology, Pittsburgh Center for Evolutionary Biology and Medicine, University of Pittsburgh School of Medicine, Pittsburgh, PA 15213, USA

**Keywords:** beneficial, normalization method, genetic screen, phenomics, microbiology

## Abstract

Microbial growth characteristics have long been used to investigate fundamental questions of biology. Colony-based high-throughput screens enable parallel fitness estimation of thousands of individual strains using colony growth as a proxy for fitness. However, fitness estimation is complicated by spatial biases affecting colony growth, including uneven nutrient distribution, agar surface irregularities, and batch effects. Analytical methods that have been developed to correct for these spatial biases rely on the following assumptions: (1) that fitness effects are normally distributed, and (2) that most genetic perturbations lead to minor changes in fitness. Although reasonable for many applications, these assumptions are not always warranted and can limit the ability to detect small fitness effects. Beneficial fitness effects, in particular, are notoriously difficult to detect under these assumptions. Here, we developed the linear interpolation-based detector (LI Detector) framework to enable sensitive colony-based screening without making prior assumptions about the underlying distribution of fitness effects. The LI Detector uses a grid of reference colonies to assign a relative fitness value to every colony on the plate. We show that the LI Detector is effective in correcting for spatial biases and equally sensitive toward increase and decrease in fitness. LI Detector offers a tunable system that allows the user to identify small fitness effects with unprecedented sensitivity and specificity. LI Detector can be utilized to develop and refine gene–gene and gene–environment interaction networks of colony-forming organisms, including yeast, by increasing the range of fitness effects that can be reliably detected.

## Introduction

Colony-based high-throughput screens (CBHTS) of microbes are increasingly used for basic science biomedical and industrial research (Priola *et al.* 2016; Rallis and Bahler 2016; Saleski *et al.* 2019; Zeng *et al.* 2020). These screens involve growing manually or robotically “pinned” grids of microbial colonies on agar plates and recording colony growth using imagery. The images are computationally analyzed to generate a quantitative output of colony size, which is used as a proxy for the organism’s fitness. The wide availability of tools to conduct and analyze CBHTS, combined with the growing number of artificial gene constructs for microbial model organisms, has provided a large-scale controlled approach to experimentally determine the effects of genetic and environmental perturbations on the fitness of an organism. CBHTS have been used to explore genetic interactions (Roguev *et al.* 2008; Costanzo *et al.* 2010), protein–protein interactions (Becker *et al.* 2004; Kamiya *et al.* 2010), chemical–genetic interactions (Parsons *et al.* 2004, 2006; Galardini *et al.* 2019), and microbial pathogenicity (Butland *et al.* 2008).

CBHTS fast track discovery thanks to the scale at which they are performed. Various colony growth characteristics such as colony size at saturation, growth rate, colony shape, opacity, color, or volume have been used as a proxy for fitness (Collins *et al.* 2006; Baryshnikova *et al.* 2010; Dittmar *et al.* 2010; Lawless *et al.* 2010; Levin-Reisman *et al.* 2010, 2014; Wagih *et al.* 2013; Young and Loewen 2013; Bean *et al.* 2014; Wagih and Parts 2014; Bischof *et al.* 2016; Zackrisson *et al.* 2016; Herricks *et al.* 2017). Of these, colony size at saturation is the most commonly used growth characteristic in CBHTS (Supplementary Table S1). However, spatial biases-like edge effects (Wagih *et al.* 2013; Bean *et al.* 2014), local competition (Wagih *et al.* 2013; Young and Loewen 2013), batch effects (Baryshnikova *et al.* 2010; Wagih *et al.* 2013), source-based bias (Bean *et al.* 2014), light artifacts (Dittmar *et al.* 2010; Lawless *et al.* 2010), agar surface nutrient heterogeneity (Wagih *et al.* 2013; Young and Loewen 2013; Bean *et al.* 2014) and humidity (Bean and Ideker 2012), all lead to undesired colony size differences that are not relevant to the biological question being investigated (Supplementary Figure S1). These spatial biases need to be corrected before making any biological inferences. The extent of spatial bias is difficult to predict *a priori*, making its identification and correction a substantial computational challenge (Baryshnikova *et al.* 2010). A variety of existing tools implement normalization algorithms to correct for spatial biases, including the HT colony grid analyzer (Collins *et al.* 2006), Colonyzer (Lawless *et al.* 2010), ScreenMill (Dittmar *et al.* 2010), ScanLag (Levin-Reisman *et al.* 2010), SGATools (Wagih *et al.* 2013), Balony (Young and Loewen 2013), Scan-o-matic (Zackrisson *et al.* 2016), and MATLAB Colony Analyzer Toolkit (MCAT) (Bean *et al.* 2014) (Supplementary Table S1).

Most of the existing tools rely on the following assumptions about the distribution of fitness effects (DFE): that the colony sizes in an experiment are normally distributed, and that genetic manipulations rarely cause significant fitness deviation from wildtype (Wagih *et al.* 2013). These assumptions can be violated in experiments with biased sets of mutants (Baryshnikova *et al.* 2010; Herricks *et al.* 2017), or experimental conditions producing a high variance in the DFE (Baryshnikova *et al.* 2010). Even in unbiased genome-scale screens, the assumption of normal distribution is usually violated due to a skew toward negative fitness effects (Wloch *et al.* 2001; Joseph and Hall 2004; Van den Bergh *et al.* 2018). As a result, while existing methods can reliably detect large changes in fitness, they are less sensitive in detecting small effects that are difficult to differentiate from noise. This difficulty in detecting small fitness effects is especially pronounced for small increases in fitness. Overall, methods that rely on strict assumptions about the underlying DFE reduce the power of CBHTSs for broader scientific inquiry.

Here, we present the linear interpolation-based detector (LI Detector or LID), a CBHTS framework designed to avoid making any *a priori* assumptions about the underlying DFE. This two-part, experimental, and analytical framework utilizes a reference colony grid on every plate of the experiment to predict and correct for spatial biases. The reference grid is an isogenic population of colonies that are evenly distributed over the agar surface to act as internal local controls (Zackrisson *et al.* 2016; Vakirlis *et al.* 2020). Our results show that the LI Detector’s reference colony-based linear interpolant can successfully control for spatial bias. LI Detector is a tunable system that can provide the users with the ability to identify 5% or lower fitness effects with very high specificity and sensitivity. LI Detector performs as well as a popular existing method, MCAT (Bean *et al.* 2014), when the underlying DFE is normal and better when that is not the case.

## Materials and methods

### Validation experiment using an isogenic population

A method validation experiment was conducted using an isogenic population that was mocked as either references or mutants.

#### Yeast strain, medium and robotic equipment

A previously characterized prototrophic *Saccharomyces cerevisiae* strain in the S288C background, FY4 (Winston *et al.* 1995), was used to conduct experiments in YPD medium (1% w/v yeast extract, 2% w/v peptone, 2% w/v dextrose, and 2% agar in the case of solid medium). A single colony of FY4 (Winston *et al.* 1995) selected from a streak out was used to inoculate liquid YPD medium and grown overnight at 30°C. This culture was used to create four 384-well glycerol stocks with wells containing 18 µL of 50% glycerol and 42 µL of culture media. Two to five wells in each stock were left empty to create gaps in the colony grid when pinned on solid medium. The stocks were stored at −80°C before use. The benchtop RoToR HDA robotic plate handler (Singer Instruments Co Ltd, Roadwater, UK.) was used for plate-to-plate cell transfer (Supplementary Table S2).

#### Pin-copy-upscale

**Figure 1:**
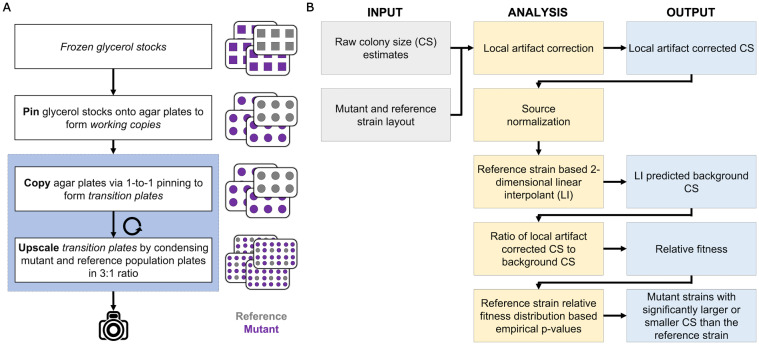
The LI Detector framework consists of integrated experimental and analytical pipelines. (A) The pin-copy-upscale experimental pipeline from frozen glycerol stocks (top) to imaging (bottom). Each box represents a pinning step, and the steps within the sky-blue highlighted portion can be repeated until the desired colony density is reached. Illustrations to the right of the flowchart is a simplified representation of four experimental plates. A reference population (gray) is introduced on every plate during the first upscale step. The analytical pipeline uses this population for spatial bias correction and relative fitness estimations for the mutant strains of interest (purple). (B) Workflow of the analysis pipeline where columns from left to right represent user inputs, analytical steps, and outputs. User inputs consist of raw colony size estimates and the strain layout of the plates. The analytical pipeline performs: (1) local artifact correction, (2) source normalization, (3) reference-based background colony size estimation using a 2-dimensional linear interpolation, (4) corrects for spatial bias by dividing the local artifact corrected colony sizes with the background colony sizes and provides a measure of relative fitness, and (v) assigns empirical *P*-values using the reference strain relative fitness distribution. The outputs include local artifact corrected colony sizes, background colony sizes, spatially corrected relative fitness, and mutant strains identified as having a mean colony size that is significantly larger or smaller than the reference strain.

The LI Detector experimental pipeline follows a pin-copy-upscale protocol when starting from frozen glycerol stocks. The copy-upscale steps are repeated until the desired colony density is reached (Figure 1A). The four glycerol stock plates were pinned at 384-density to generate working copy agar plates. This process was performed using the RoToR HDA robot with default settings (Supplementary Table S2). The working copies were incubated at 30**°**C for 60 h to reach saturation. These were then copied 1-to-1 to make transition plates (#1) using default RoToR HDA settings (Supplementary Table S2) and incubated at 30**°**C for 48 h. Distinct combinations of the four transition plates (#1) were then condensed to make four 1536-density upscale plates (#1) using default RoToR HDA settings (Supplementary Table S2). The distinct combinations ensure that colony grids from each plate occupy different positions on the four higher density plates. The upscale plates (#1) were incubated at 30**°**C for 30 h, after which they were copied 1-to-1 to transition plates (#2) using custom RoToR HDA settings (Supplementary Table S2). The overshoot setting value at the target plate was increased, from 2  to 2.5 mm, to compensate for the agar surface's unevenness and the smaller pin size of the higher density pin pads. These plates were incubated at 30**°**C for 30 h. Four 6144-density upscale plates (#2) were then made by condensing the four transition plates (#2) in distinct combinations using default RoToR HDA settings (Supplementary Table S2). These were incubated at 30**°**C until they reached saturation and imaged at the following eleven time points: 1.0, 1.4, 2.9, 4.0, 4.9, 6.1, 6.9, 7.8, 9.0, 10.0, 11.0 h (Supplementary Figure S2). All images are available at https://pitt.box.com/s/xbchjoa4ta3oq2g50q4avfypjrgz7poq.

For the purposes of evaluating the performance of LI Detector, colonies originating from a random working copy were mocked as reference strains, while the colonies from the other three working copies were mocked as mutant strains. In the upscale plates (#2) used for our analyses, one-fourth of all colonies correspond to references, and the rest are treated as mutants. These plates had 16 technical replicates for every colony that was present in the working copy. Supplementary Figure S3 provides a simplified visual representation of the plates at all pinning stages.

### Colony size estimation

Raw estimates of colony sizes are an input to the LI Detector framework (Figure 1B) and can be obtained in the user’s manner of choice. Here, a custom-made lightbox with an overhead camera mount was built to acquire high-resolution images using a commercially available SLR camera (18Mpixel Rebel T6, Canon USA Inc., Melville, NY, USA). The 6144-density upscale plates (#2) were imaged at eleven time points beginning right after pinning until the colonies reached saturation, around 11 h later (Supplementary Figure S2). Saturation was determined as the point at which the colonies would touch each other if the plates were incubated for any longer. The images were analyzed in bulk using the “analyze_directory_of_images()” function of the MCAT (Bean *et al.* 2014) with the default threshold parameter (1.25) to provide colony size estimations (https://github.com/sauriiiin/lidetector/blob/master/imageanalyzer.m). The output files containing colony size information along with the images is available at https://pitt.box.com/s/xbchjoa4ta3oq2g50q4avfypjrgz7poq.

### Spatially cognizant colony size database

A unique position identifier (pos) was given to every possible colony position across the different plates of the experiment. Each pos was linked to plate density, plate number, column number, row number and stored in a “position to coordinate” table (pos2coor). A “position to mutant name” table (pos2orf_name) was used to store information on which colony position was occupied by which mutant. The colony size estimations and the pos2coor table were used to store the colony sizes in a spatially cognizant manner. Supplementary Figure S3 is a visual representation of the plate maps made using the pos2coor and pos2orf_name tables. The colonies’ spatial layout and identity are an input to the LI Detector framework (Figure 1B) and should be provided in this format by users. The format, along with the data collected for this manuscript, is available at https://github.com/sauriiiin/lidetector.

### LI Detector analytical pipeline

The LI Detector analytical pipeline (Figure 1B, https://github.com/sauriiiin/lidetector/blob/master/lid.m) is designed to make fitness assessments using local reference colony information.

#### Border colony removal

Border colonies tend to grow larger because of increased access to nutrients (Baryshnikova *et al.* 2010; Wagih *et al.* 2013; Young and Loewen 2013; Bean *et al.* 2014; Zackrisson *et al.* 2016). To remove this artifact, we ignore colony size estimations of one, two, and four border rows and columns from 384, 1536, and 6144-density plates, respectively. Doing this resulted in 4864 colonies for 304 mock references and 14,576 colonies for 911 mock mutants across four 6144-density plates. All further analysis is done using this set.

#### Local artifact correction

Local artifact correction (AC) is inspired by the “competition correction” feature present in existing tools (Wagih *et al.* 2013; Young and Loewen 2013). An “artifact score” is assigned to every colony on a plate as a ratio of its colony size compared to its current and past neighbors. The current neighbors are a colony’s eight immediate neighbor colonies, and the past neighbors are eight neighboring colonies that were pinned from the same source plate. The reference population’s artifact scores are used to determine outliers, defined as two median adjusted deviations (MADs) or more from the median. Outliers are defined as colonies growing disproportionately big or small as compared to their neighboring colonies. Outliers that occur as a localized group of three or more neighbors of both big and small colonies are considered for correction. The less abundant outlier in the group is expected to have driven the phenotype. For example, a single small or dead colony would increase the relative access to nutrients for all its neighbors, which would all be expected to grow bigger than usual and vice-versa. Raw colony sizes of all the driver’s immediate neighbors are median normalized using the median reference population colony size for the plate. Users have the option to skip this correction.

#### Source normalization

LI Detector uses a source-based computational deconstruction of high-density plates into their four lower density sources to correct the source-related colony size differences introduced during the upscaling process (Supplementary Figures S4 and S5). This correction is a reimplementation of MCAT’s interleave filter (Bean *et al.* 2014). Each source-deconstruct is individually normalized in the later steps, making it necessary for the penultimate density plates to have a reference population grid. Users have the option to skip this correction, although we strongly recommend against skipping if upscales are performed.

#### Reference-based normalization

A two-dimensional linear interpolant is applied to the reference population grid to estimate expected colony sizes on the entire colony grid, including the reference colony positions. This reference colony-based estimated colony size is referred to as the “background colony size.” The background colony sizes represent the predicted reference colony growth on every position of the grid conditioned upon the spatial context. Relative fitness is estimated as the ratio of the local artifact corrected colony size to the background colony size, thus controlling for spatial context.

The goal of the two-dimensional linear interpolation is to predict the unknown function f at the point (x, y). It is assumed that the value of f is known at the four points $Q_{11}=\left(x_{1},\;y_{1}\right)$, $Q_{12}=\left(x_{1},\;y_{2}\right)$, $Q_{21}=\left(x_{2},\;y_{1}\right)$, and $Q_{22}=\left(x_{2},\;y_{2}\right)$. The first step is to conduct linear interpolation in the x-direction:

$$f\left(x,\;y_{1}\right)\approx \;\frac{x_{2}-x}{x_{2}-\;x_{1}}\;f\left(Q_{11}\right)+\;\frac{x-\;x_{1}}{x_{2}-\;x_{1}}\;f\left(Q_{21}\right)$$

$$f\left(x,\;y_{2}\right)\approx \;\frac{x_{2}-x}{x_{2}-\;x_{1}}\;f\left(Q_{12}\right)+\;\frac{x-\;x_{1}}{x_{2}-\;x_{1}}\;f\left(Q_{22}\right).$$

The next step is to move in the y-direction to obtain the desired function:

$$f\left(x,y\right)\approx \;\frac{y_{2}-y}{y_{2}-\;y_{1}}\;f\left({x,y}_{1}\right)+\;\frac{y-\;y_{1}}{y_{2}-\;y_{1}}\;f\left(x,y_{2}\right).$$

The same result will be obtained if the interpolation is done first along the y direction and then along the x direction.

### Different strategies for fitness estimation

The LI Detector analytical pipeline is applied to colony size estimates to control for spatial bias and measure relative fitness as described above. The analytical pipeline is used as-is (LID), without local artifact correction (LID-AC), and without source-normalization (LID-SN) to measure the impact of these components on the downstream analysis. Raw observed colony size estimates were also used as “fitness” measurements without performing any normalization (NO-NORM). Fitness estimates were also made using the MCAT’s (Bean *et al.* 2014) SpatialMedian normalization with window size nine along with the Interleave filter (https://github.com/sauriiiin/sau-matlab-toolkit/blob/master/image2resBEAN.m).

### Measuring spatial bias and the accuracy of background colony size

The coefficient of variance of fitness and colony size estimations was used to measure the impact of spatial bias in colony sizes of an isogenic population (Supplementary Figure S6A). Ten random observations were picked, with replacement, 2000 times to measure the coefficient of variance as a percentage of the mean (CV%). CV% distributions for LID, LID-AC, LID-SN, MCAT (Bean *et al.* 2014), and NO-NORM were compared using the Wilcoxon rank-sum test.

The accuracy of background colony size was measured using root mean square error (RMSE) estimation as a percentage of the average observed colony size (Supplementary Figure S6B). A random colony size predictor (RND) was used as a null model for background colony size prediction. The RND generated random colony sizes from a normal distribution, with the *rnorm* function in R (R Core Team 2013) using the mean and standard deviation of observed colony sizes. The Wilcoxon rank-sum test was used to compare RMSE results from LID, LID-AC, LID-SN, MCAT (Bean *et al.* 2014), and RND.

### Calculating significant fitness changes and assigning phenotypes

The relative fitness of each strain was measured as the mean of estimated relative fitness among its replicates. This measurement was done after removing the outlier observations based on three MAD. MAD is a more robust outlier removal technique than other measures such as mean, standard deviations or z-scores, because it does not assume a normal distribution, is not impacted by outliers and is capable of detecting outliers in small samples (Iglewicz and Hoaglin 1993). The reference strain relative fitness distribution was used as a null distribution for hypothesis testing, as the reference strains are isogenic, and no real fitness differences are expected. An empirical *P*-value was estimated for all mutant strains based on where they fall relative to this null distribution (https://github.com/sauriiiin/lidetector/blob/master/lid.m). For example, an empirical *P*-value of 0.05 or below would mean that the mutant’s relative fitness is in the top or the bottom 2.5th percentile of the reference fitness distribution. The phenotype of mutant strains significantly different from the reference population is classified as “beneficial” or “deleterious,” depending on whether its estimated relative fitness is above or below 1. The remaining mutant strains that do not have a significant change in fitness are classified as having a “neutral” phenotype.

### Alternate strategies for detecting significant fitness changes

To ensure that our results were not confounded by using the same strain for spatial bias correction and for empirical testing, we mocked colonies originating from a working copy other than the one chosen as the reference as a “tester” population. Hence, the upscale plates (#2) in this case had one-fourth of all colonies as references used for spatial bias correction, another one-fourth as testers used for empirical testing and the remaining half were treated as mutants. Everything else being the same, the null distribution for the empirical testing was determined using the relative fitness distribution of the tester. Results from this analysis (https://github.com/sauriiiin/adaptivefitness/blob/master/scripts/paper/FIGURES_REV.R) are shown in Supplementary Figure S7.

To ensure that our results were robust to the statistical test being performed, the relative fitness distributions, post outlier removal, for every mutant, were compared to that of the reference strain using the nonparametric Wilcoxon ranksum test (Wilcoxon 1946; Mann and Whitney 1947). The resultant *P*-values were corrected for multiple hypothesis testing using *Q*-value estimations as defined in Storey (2002). A *Q*-value cut-off of 0.05 was used to determine significant fitness deviations. Results from this analysis (https://github.com/sauriiiin/adaptivefitness/blob/master/scripts/paper/FIGURES_REV.R) are shown in Supplementary Figure S8.

### Empirical strategy for performance evaluation

**Figure 2:**
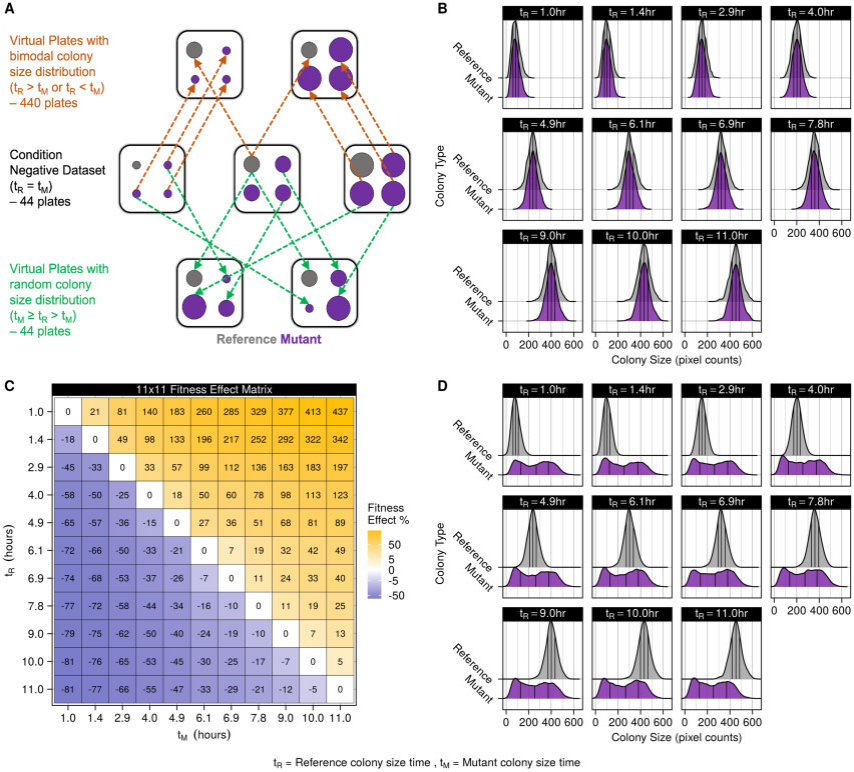
Condition negative and positive datasets used for performance evaluation. (A) Illustration of the condition positive and negative datasets described in Table 1. The squares represent plates, and the circles within them represent colonies. The reference colonies are colored as gray and the mutant colonies as purple. The middle row represents the condition negative dataset shown as a single plate at three different time points. There were 44 plates in this dataset. The top row shows two virtual plates made by combining reference colony size data from one time point with mutant colony size data from another. These virtual plates had a bimodal colony size distribution. There were 440 such virtual plates. The bottom row shows two virtual plates where the reference colony size data taken from one time point is combined with mutant colony size data randomly selected from any time point. These virtual plates had a random colony size distribution. There were 44 such virtual plates. All virtual plates maintain the same spatial layout of colonies as the condition negative dataset, as is shown by the arrows. *t_R_* is reference colony size time, and *t_M_* is mutant colony size time. (B) Reference and mutant population colony size density plots from the condition negative dataset. Vertical black lines within the density plots represent the lower, middle, and upper quartile. All mutants are expected to have a neutral phenotype. (C) Fitness effect matrix of the condition positive virtual plates with bimodal colony size distribution. Mutant (t_M_) and reference colony size time (*t_R_*) is represented on the *x*-axis and *y*-axis, respectively. The fitness effect was calculated as the difference in mutant and reference mean colony sizes as a percentage of the reference mean colony size (Supplementary Figure S10). This dataset was used to calculate the sensitivity of the LI Detector as a function of the fitness effect. (D) Reference and mutant population colony size density plots from the condition positive virtual plates with random colony size distribution. Vertical black lines within the density plots represent the lower, middle, and upper quartile. Mutant strains could be beneficial, deleterious, or neutral. These virtual plates were used to evaluate LI Detector's sensitivity in situations where *a priori* assumptions of fitness are challenging to make.

An empirical strategy was devised to thoroughly examine the LI Detector’s performance. A condition negative and positive dataset were created to estimate specificity and sensitivity, respectively (Table 1, Figure 2A, Supplementary Figure S9).

**Table 1:** Empirical strategy for performance evaluation

Test Dataset	Time of colony size data	Colony size distribution	Expected phenotype	Performance measure
**Condition Negative**	*t_R_* = *t_M_*	Uniform	Neutral	Specificity
**Condition Positive (Virtual Plates)**	*t_R_* > *t_M_*	Bimodal	Deleterious	Sensitivity
	*t_R_* < *t_M_*		Beneficial	
	*t_M_*≥ *t_R_* > *t_M_*	Random	Deleterious Neutral Beneficial	

*t_R_* = Reference colony size time, *t_M_* = Mutant colony size time

The testing space consists of a condition negative and condition positive datasets. The colony size datasets are generated using an isogenic population of *S. cerevisiae* grown on four 6144-density agar plates (see *Materials and Methods*). These plates were imaged at eleven time points from pinning to saturation. A subset of colonies on the plates were mocked as references, and the rest were mocked as mutants. This dataset was considered condition negative, as the reference and mutant colonies: (1) are isogenic, and (2) grown to the same time point. The condition positive dataset was made up of virtual plates created by combining reference and mutant colony size data from different time points so that the DFE is either bimodal or random. These datasets are used to measure the ability of the LI Detector to observe a variety of fitness effects. *t_R_* represents the reference colony size time, and *t_M_* the mutant colony size time.

The condition negative dataset consisted of data where the mock mutants and references have similar colony size distribution. To this end, colony size data taken from any time point represents a unique condition negative dataset (Figure 2B). We tested 44 such plates, four plates for the 11 time points that images were taken. The proportion of mock mutant strains that are successfully called neutral by the LI Detector represents the true negative rate or specificity.

The condition positive dataset consisted of colony size data where mutant strains can be deleterious or beneficial. Two sets of virtual plates were created to generate such a condition positive dataset. The first set of virtual plates contained a bimodal distribution of colony sizes (Supplementary Figures S10 and S11) where colony size estimations for reference and mutant colony positions came from two different time points while maintaining their topological context (https://github.com/sauriiiin/paris/blob/master/techPowA.m). The fitness effect between the reference and mutant colony size distribution is the difference of their mean colony sizes as a percentage of the mean reference colony size (Figure 2C). We tested 440 virtual plates with bimodal colony size distribution resulting from combining reference colony size data from 11 time points (*t_R_*) with mutant colony size data taken from 10 time points (*t_M_*) and having four plates for each *t_R_*_—_*t_M_* combination.

The second set of virtual plates contained a random distribution of colony sizes where reference colony size data from a particular time point was combined with mutant colony size data randomly selected from all time points (Figure 2D). Colony size estimates for replicates of the same mutant were all selected from the same time point (https://github.com/sauriiiin/adaptivefitness/blob/master/scripts/4CX/4C_MESSUP.R). We tested 44 virtual plates with random colony size distribution by having 4 plates for the 11 time points that reference colony size data (*t_R_*) can be taken from (Figure 2D).

**Figure 3:**
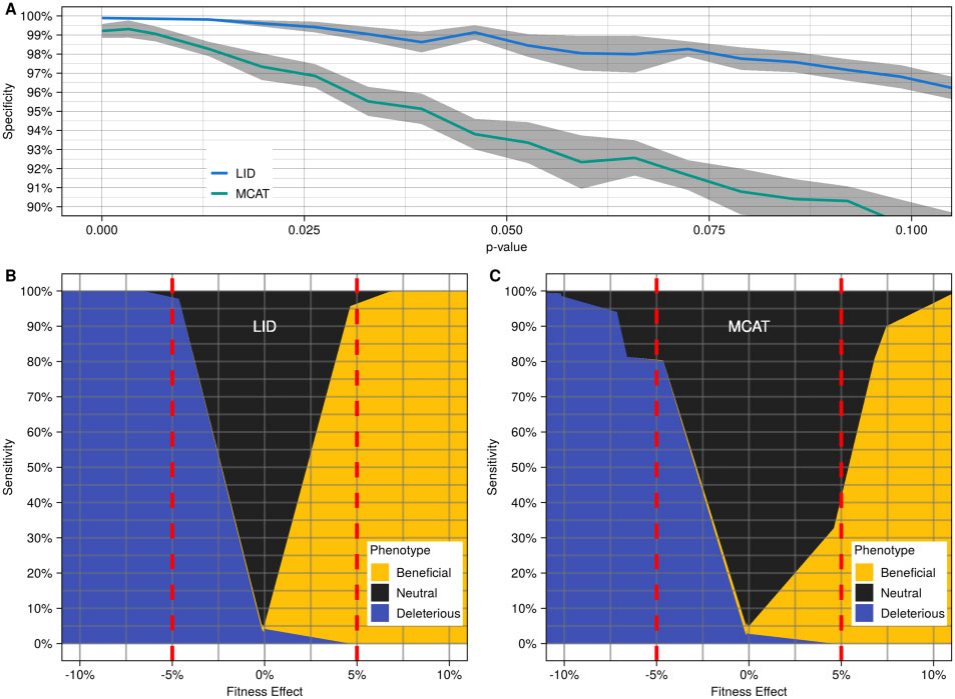
The LI Detector has high specificity and sensitivity. (A) Average specificity (solid colored line) and standard error (gray region) at various empirical *P*-value cut-offs for LID (blue) and MCAT (Bean *et al.* 2014) (green). Empirical *P*-values (*x*-axis) calculated using the reference strain relative fitness distribution (see *Materials and Methods*). Specificity (*y*-axis) was estimated using the condition negative dataset as the proportion of mutants classified as neutral (see *Materials and Methods*). (B) LID phenotype classification results from the virtual plates with bimodal distribution are arranged according to increasing fitness effects. Here, the fitness effect is the mean mutant and mean reference colony size difference as a percentage of the reference colony size for each virtual plate. Sensitivity is calculated as the proportion of mutants correctly identified as significantly different (beneficial or deleterious) than the reference for each fitness effect value. The dotted red line indicates a 5% fitness effect. A 5% false positive rate was maintained while generating these results. (C) MCAT (Bean *et al.* 2014) phenotype classification results from the same data as (B).

**Figure 4:**
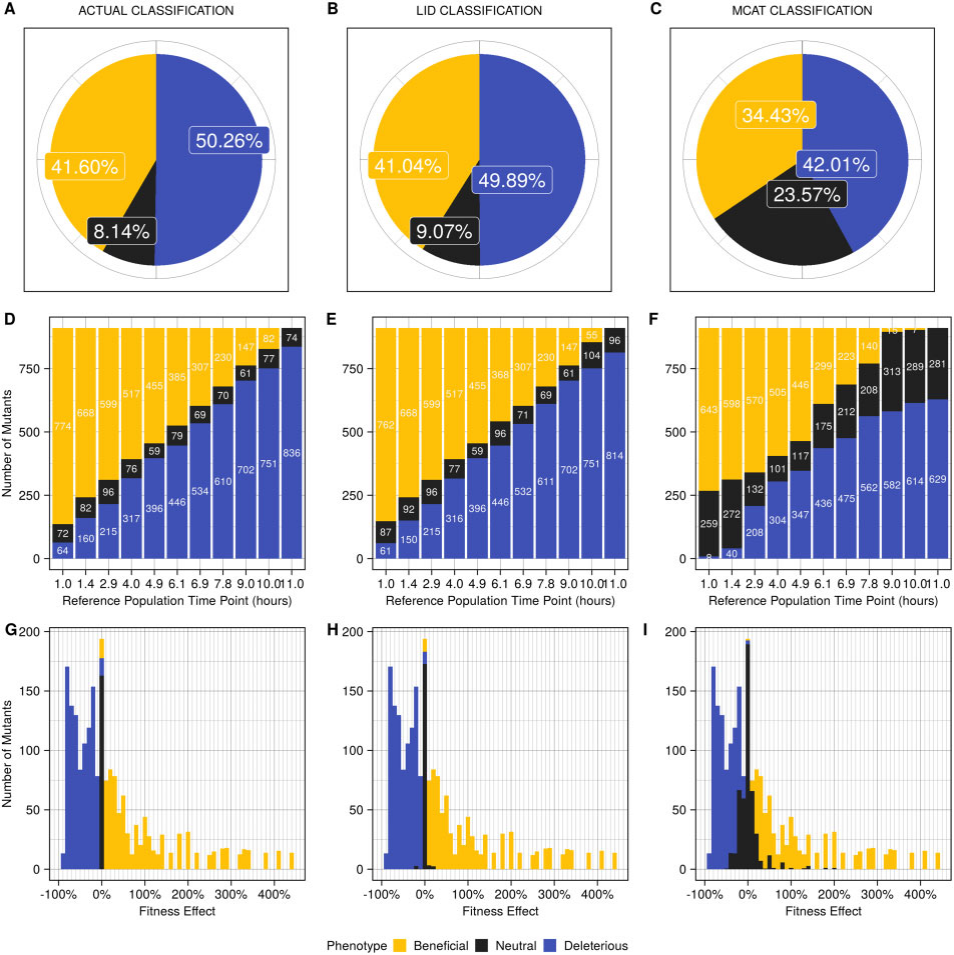
The LI Detector maintains high sensitivity even when the underlying DFE is random. (A) The actual classification of the mutants in the random DFE condition positive dataset, per construction, with 41.60% beneficial, 50.26% deleterious, and 8.14% neutral. (B) and (C) show the classification results from LID and MCAT (Bean *et al.* 2014), respectively. (D–F). Bar graphs showing (D) actual, (E) LID, and (F) MCAT (Bean *et al.* 2014) classification of mutants for each virtual plate with random DFE. The virtual plates are arranged according to their reference colony time point. (G–I) Bar graph of pooled results from all plates arranged according to the fitness effects for the (G) actual classification, (H) LID and (I) MCAT (Bean *et al.* 2014). Each bar has a width of 10%. False positive rate was maintained at 5% for both LID and MCAT (Bean *et al.* 2014) in these analyses.

Mutants that are successfully called beneficial or deleterious in these virtual plates are used to estimate the true positive rate or sensitivity of the LI Detector (https://github.com/sauriiiin/adaptivefitness/blob/master/scripts/4CX/4C_POWDY.R). For the virtual plates, an empirical *P*-value cut off that controls the false positive rate at 5% was used to make the examination of sensitivity comparable between LID, LID-AC, LID-SN, MCAT(Bean *et al.* 2014), and NO-NORM. The results from this analysis are represented in Figures 3 and 4, Supplementary Figures S12 and S13.

### Measuring the impact of the number of references and replicates

**Figure 5:**
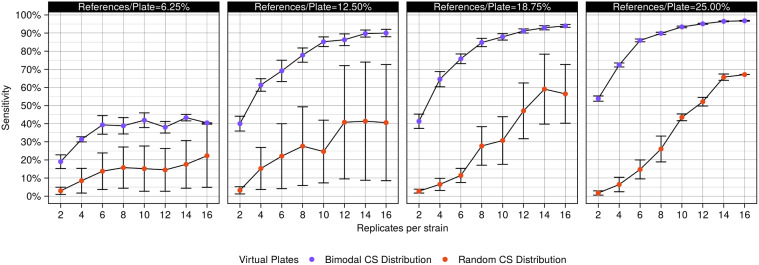
Sensitivity is directly related to the number of references and replicates. Sensitivity for observing 5% fitness effects, as a function of the varying proportion of references per plate (individual panels) and the number of replicates per strain (*x*-axis) was estimated for virtual plates with bimodal (purple) and random (orange) colony size distribution. Error bars represent a single standard deviation.

The reference colony proportion was sequentially reduced from 25% to 18.75% to 12.5% to 6.25% by masking one-fourth of the existing reference grid each time. This reduction was achieved by masking colonies on the 384-density mock reference plate and then propagating those masked colonies through the other densities. The number of replicates per strain was reduced in parallel by masking the *n*th replicate of every mock strain. Replicates were reduced from 16 to 2 in increments of 2. This process was repeated ten times to mask a variety of replicate combinations (https://github.com/sauriiiin/paris/blob/master/techPowA.m). Results from the analysis of the resultant plates are represented in Figure 5, Supplementary Figures S14 and S15.

### Data availability

All data generated/analyzed in this study is available in the main text, in the Supplementary Figures and Tables, and as Supplementary Data files. All supplementary data are also on GitHub: https://github.com/sauriiiin/lidetector. Supplementary material is available at figshare: https://doi.org/10.25387/g3.13373255.

### Code availability

The code is available to download at https://github.com/sauriiiin/lidetector, along with instructions on how to use it. Image processing, relative fitness estimations, and analyses presented in the result section are available at https://github.com/sauriiiin/sau-matlab-toolkit. All images within the main article and supplementary data were generated using code available at https://github.com/sauriiiin/adaptivefitness/tree/master/scripts/paper.

Detailed protocol on the use of LI Detector can be found at https://www.protocols.io/private/21D4D9D12A7E11EBAB590A58A9FEAC2A.

## Results

### Development of a new CBHTS framework

The LI Detector framework is specifically designed to correct spatial bias and sensitively detect small but significant fitness changes without making *a priori* assumptions about the underlying DFE of tested strains (thereafter, “mutant” strains). The experimental pipeline (Figure 1A) follows a pin-copy-upscale protocol that serves two purposes. It reduces colony size differences that arise during the pinning process and adds a reference colony grid (Zackrisson *et al.* 2016) on every plate. The copy step is instrumental in reducing the source-based bias (Bean *et al.* 2014) introduced after upscaling (Supplementary Figure S5). The analytical pipeline utilizes the reference colony grid to correct spatial bias and infer the fitness of mutant strains relative to the reference strain. The analytical pipeline (Figure 1B) consists of five main steps: (1) local artifact correction (AC), (2) source normalization (SN), (3) reference strain based background colony size estimation using a 2-dimensional linear interpolant, (4) estimation of spatially-corrected relative fitness as the ratio of the local artifact corrected colony sizes divided by the estimated background colony sizes, and (5) empirical hypothesis testing to identify mutant strains with colony size distributions that have a significantly larger or smaller mean than the reference strain.

The local AC step is designed to reduce spatially localized colony size differences that arise due to differential access to nutrients. It is similar to the competition correction feature implemented by several existing methods (Wagih *et al.* 2013; Young and Loewen 2013). The SN step controls for differences in colony sizes that occur due to the upscaling process. This step was reimplemented from the interleaving feature of MCAT (Bean *et al.* 2014). Briefly, it deconstructs the colony size estimates of the higher density plates into subsets corresponding to the source plates used for the upscaling (Supplementary Figure S4). Both local AC and SN are provided as optional steps in the LI Detector analytical pipeline.

The background colony size estimation step predicts the size that a reference colony would be for every position on the plate. This step employs a two-dimensional linear interpolant based on the reference colony grid. Relative fitness is then assigned to every colony as a ratio of the local artifact corrected colony size to the predicted background colony size. This estimate of relative fitness corrects local spatial bias without making any assumptions of the underlying DFE. The only assumption is that, for any location on the plate, the spatial bias is expected to affect the reference and mutant colonies to an equal extent.

Each mutant strain is assigned a relative fitness value corresponding to the average relative fitness of its replicate colonies. The distribution of relative fitness estimates for the reference strain is then used as null distribution to calculate empirical *P*-values describing the probability of the reference strain having a more extreme value of relative fitness than the mutant strain. The empirical *P*-values are used to determine the significance of the mutant strain fitness deviation from the reference strain (see *Materials and Methods*).

In what follows, we compare the performance of LI Detector with one of the most versatile and robust tools available for correcting spatial bias, MCAT (Bean *et al.* 2014). The overall workflow adopted for testing the two methods’ performance is described in Table 1 and Supplementary Figure S9. In brief, we estimated the specificity and sensitivity of the LI Detector and MCAT using colony size datasets generated using an isogenic population of *S. cerevisiae* (see *Materials and Methods*). A subset of colonies was mocked as references, and the rest were mocked as mutants. The LI Detector and MCAT (Bean *et al.* 2014) spatial bias correction was applied independently. For consistency, our empirical *P*-value calculation strategy was used for the two methods. The mutant strains were classified into beneficial, deleterious, or neutral phenotypes depending on whether their relative fitness was significantly higher, significantly lower, or unchanged compared to the reference distribution.

### Construction of condition negative and positive datasets for performance evaluation

To evaluate the LI Detector's performance, we constructed datasets where the underlying DFE was known, but colony sizes were realistically affected by spatial biases and other technical artifacts of CBHTS (Figure 2A). To this end, we applied the pin-copy-upscale experimental pipeline of our framework (Figure 1A), starting with four 384-well glycerol stock plates, each containing replicate frozen cultures of the same strain (FY4, Winston *et al.* 1995). This procedure generated four 6144-density agar plates containing 16 replicate colonies for each culture in the starting glycerol stock plates (see *Materials and Methods*). The sizes of these colonies were measured at eleven time points while they grew to saturation (Figure 2B, Supplementary Figure S2). The colonies originating from one of the glycerol stock plates were treated as reference, and the rest were treated as mutants.

To estimate specificity, we assembled a “condition negative” dataset consisting of colony size measurements of our plates at eleven time points. None of the mutants in this dataset should be significantly larger (beneficial) or smaller (deleterious) than the references (Figure 2B). We then assembled two artificial “condition positive” datasets consisting of “virtual plates” that we used to evaluate the sensitivity of the LI Detector (Figure 2, C and D). These virtual plates were constructed so that the underlying DFE would be known and readily comparable to the LI Detector and MCAT (Bean *et al.* 2014) results. The first condition positive set combined colony size estimates of the mock references and mutants from two different time points, resulting in virtual plates with bimodal colony size distributions: a reference distribution, and a mutant distribution with a smaller or larger mean (Supplementary Figure S10). The fitness effect was measured as the difference in the mean colony sizes of the two distributions as a percentage of the reference distribution mean colony size (Figure 2C). Doing this allowed us to evaluate sensitivity for a broad range of fitness effects. The second condition positive set combined the reference distribution from a single time point with mutant colony sizes from randomly chosen time points, resulting in virtual plates with random DFE (Figure 2D). The random DFE allowed us to estimate sensitivity when the traditional assumptions used for spatial bias correction are unwarranted. It is important to note that all virtual plates retain realistic spatial biases in colony sizes because they maintain the original plate layout.

We leveraged the condition negative and positive datasets to compare the performance of LI Detector (LID), LI Detector without source normalization (LID-SN), and LI Detector without local artifact correction (LID-AC) with that of MCAT (Bean *et al.* 2014). We also used a random generator (RND) to assign background colony sizes by only taking the global colony size distribution of the reference population into account. Lastly, the observed colony sizes were used as-is, as “fitness” estimates to generate phenotype results when no normalization (NO-NORM) was done on the datasets.

### LI Detector can accurately estimate background colony sizes and eliminate spatial bias

In our condition negative dataset, variation in colony sizes should only stem from the spatial bias and from occasional nongenetic biological variation expected for some isogenic populations exhibiting clonal heterogeneity (Van Egeren *et al.* 2018). Hence, the fitness estimates obtained after spatial bias removal should only reflect biological variability and the added noise from the bias removal process. We measured the coefficient of variance percentage (CV%) of the colony sizes and fitness estimates for images taken at multiple time points (see *Materials and Methods*). LID, LID-AC, and MCAT (Bean *et al.* 2014) showed a significant reduction in CV% compared to NO-NORM, while LID-SN did not (Supplementary Figure S6A). This finding indicates that the LI Detector can reduce spatial bias and confirms that SN plays a vital role in doing so (Bean *et al.* 2014).

The LI Detector's ability to remove spatial bias depends on the accuracy with which it can estimate background colony sizes using the reference population colony sizes. We used the RMSE between background and observed colony sizes as a percentage of the mean observed colony size to measure this. LID, LID-AC, and MCAT (Bean *et al.* 2014) RMSE% were indistinguishable for the higher time points when the colonies begin to saturate (Supplementary Figure S6B). RMSE% for LID-SN was significantly higher than LID (*P* = 0.00019, Wilcoxon rank-sum test Wilcoxon 1946; Mann and Whitney 1947), again indicating the importance of performing SN (Supplementary Figure S6C). All methods performed better than RND. Overall, these findings show the LI Detector performs as well as MCAT (Bean *et al.* 2014) in eliminating spatial biases by integrating both global and local spatial contexts.

### LI Detector identifies small fitness effects with high specificity and sensitivity

To evaluate the LI Detector’s ability to detect neutral, beneficial, and deleterious fitness effects, we estimated its specificity and sensitivity using our condition negative and positive datasets, respectively (Table 1, Figure 2, Supplementary Figure S9). Specificity was calculated as the proportion of mutant strains that were correctly classified as neutral using our condition negative dataset (Figure 2B). LID's specificity was above 98% for an empirical *P*-value cut-off of 0.05 and remained above 95% when that cut-off was increased to an empirical *P*-value of 0.1. For comparison, MCAT (Bean *et al.* 2014) showed a maximum specificity of 94.5% for an empirical *P*-value cut off of 0.05 using the same dataset (Figure 3A).

Sensitivity was estimated as the proportion of mock mutant strains correctly classified as either beneficial or deleterious at a false positive rate of 5% using our condition positive dataset with bimodal fitness distribution (Figure 2C, Supplementary Figure S10). LID’s sensitivity was higher than 95% for beneficial and deleterious fitness effects of 5%, reaching 100% for fitness effects of about 7% (Figure 3B). These findings show that LID is highly sensitive in observing small fitness effects; notably, it is equally sensitive to increases and decreases in fitness. This result depended on the fitness estimation strategy, with LID performing significantly better than LID-AC, LID-SN, and NO-NORM (Supplementary Figure S12). We also performed the same analysis using MCAT (Bean *et al.* 2014). MCAT (Bean *et al.* 2014) was 80% sensitive in detecting 5% fitness decreases, and only 40% sensitive when it came to 5% fitness increases (Figure 3C). We hypothesize that MCAT’s (Bean *et al.* 2014) lower sensitivity stems from its use of a local window of surrounding mutants rather than a reference colony grid to estimate background colony size. These results show that the LI Detector displays improved sensitivity, remarkably so for beneficial effects, for the same specificity as MCAT (Bean *et al.* 2014).

We also measured LI Detector’s performance when two different strains are used for spatial bias removal and for empirical testing. To do this, we mocked a different source plate as our “Tester” strain and repeated the above analysis (see *Materials and Methods*). We found that LID’s specificity remains high, albeit marginally lower when Tester is used for empirical testing instead of the original Reference (Supplementary Figure S7A). However, in both cases, the specificity is more than 95% for an empirical *P*-value cut-off of 0.05. Similarly, LID’s sensitivity for detecting 5% fitness effects remains more than 95% with the use of Tester (Supplementary Figure S7B). These findings show that the LI Detector’s spatial bias removal and empirical testing are independent features and offers the users with additional flexibility to suit their experimental design.

### LI Detector maintains high sensitivity when the DFE is random

We designed the LI Detector to be highly sensitive regardless of the underlying DFE. To evaluate LI Detector's performance when the underlying DFE is random, we used our condition positive dataset made of forty-four virtual plates with random colony size distribution (Figure 2D). The 44 plates combined contained 41.60% beneficial and 50.26% deleterious mutants with 16 replicate colonies of each (Figure 4A). We found that LID was 98.93% sensitive, successfully identifying 98.65% of beneficial and 99.20% of deleterious mutants (Figure 4B). In comparison, MCAT (Bean *et al.* 2014) was 83.08% sensitive and successful in identifying 82.76% of beneficial and 83.40% of deleterious mutants (Figure 4C). The false positive rate was maintained at 5% for both methods. Virtual plate-wise phenotype classification results show that the actual classification (Figure 4D) is better captured by LID (Figure 4E) and that MCAT (Bean *et al.* 2014), in general, had more false negatives (Figure 4F). LID's neutral calls were mostly limited to fitness effects of 5% or smaller, whereas MCAT (Bean *et al.* 2014) neutral calls covered a wider range of fitness effects (Figure 4, G–I). That MCAT (Bean *et al.* 2014) was considerably less sensitive than LID in this scenario was not surprising since a random underlying DFE violates the assumptions of MCAT (Bean *et al.* 2014) and other existing methods.

To examine how the choice of statistical testing strategy impacted the sensitivity of LID, we conducted the above analyses using the nonparametric Wilcoxon ranksum test (Wilcoxon 1946; Mann and Whitney 1947) instead of an empirical test (see *Materials and Methods*). For both LID and MCAT(Bean *et al.* 2014), the nonparametric test's use slightly increased true positive and false positive rates relative to the empirical test (Supplementary Figure S8B). This led to a small increase in sensitivity and a small decrease in specificity for LID (Supplementary Figure S8C) and a substantial increase in sensitivity and substantial decrease in specificity for MCAT (Bean *et al.* 2014) (Supplementary Figure S8C). Notably, LID maintained more than 95% sensitivity and specificity, regardless of the statistical test used (Supplementary Figure S8C).

### The LI Detector’s sensitivity increases with an increasing number of references and replicates

LI Detector’s superior performance comes at the cost of having to integrate a reference colony grid, and therefore use a higher number of plates to screen the same number of mutant colonies. We analyzed how the number of references per plate and the number of replicates per strain affected LID’s sensitivity. To do this, we computationally masked portions of the reference colony grid and replicates, and then reanalyzed the virtual plates with bimodal and random DFE in our condition positive dataset (see *Materials and Methods*). We observed that LID’s sensitivity in detecting 5% fitness effects increased in proportion to the number of reference colonies per plate, as well as to the number of replicates per strain in both sets of virtual plates (Figure 5). Unsurprisingly, sensitivity was higher for detecting a fitness effect of 7% (Supplementary Figure S14A). Increasing the number of replicates was most powerful when there were more references on the plate (Supplementary Figure S14B). In general, the sensitivity was higher in the virtual plates with bimodal than random DFE (Figure 5). These observations are consistent with the finding that RMSE% is inversely related to the number of reference colonies per plate (Supplementary Figure S15). On the other hand, LID’s specificity was consistently above 95%, independent of the fitness estimation strategy (see *Materials and Methods*), the proportion of references per plate, and the number of replicates per mutant strain (Supplementary Figure S13). The LI Detector users may choose the number of references and replicates adequate for their purposes as a function of the fitness effects they expect to observe and the sensitivity they aim to achieve.

## Discussion

LI Detector is a CBHTS framework (Figure 1) that generates reliable and well-resolved fitness estimations without being dependent on *a priori* assumptions of the DFE (Figures 3, A and B and 4, B, E, H). LI Detector is specifically designed to observe small deleterious and beneficial fitness changes (Figure 3B). Therefore, it is a valuable method for precision phenotyping and for improving the resolution of gene–gene and gene–environment interaction networks of colony forming organisms.

Existing spatial bias correction methods work best in unbiased genome-wide studies with a large number of plates and mutants (Baryshnikova *et al.* 2010). While alternate methods have been developed to increase sensitivity at the small scale level (Herricks *et al.* 2017), LI Detector provides a flexible approach that can be applied to CBHTS independent of their scale and of the choice of strains to screen. For example, LI Detector can be used as efficiently for a highly biased screen of nonsynonymous mutations in a single gene to identify important residues (Garst *et al.* 2017; Bao *et al.* 2018; Guo *et al.* 2018; Roy *et al.* 2018; Sadhu *et al.* 2018; Sharon *et al.* 2018; Despres *et al.* 2020), or for a genome-wide synthetic genetic array used to infer genetic interactions (Costanzo *et al.* 2010, 2016; Zimmermann *et al.* 2017; Klobucar and Brown 2018; Kuzmin *et al.* 2020). LI Detector may conceivably be leveraged for high-throughput quantitative protein interaction mapping as well. This freedom of experimental design expands the applicability of CBHTS for broader scientific inquiry.

We show that LI Detector has the power to uncover significant fitness effects as small as 5% with 95% sensitivity when 25% of the plate is dedicated to reference colonies and mutant strains are represented by 16 replicate colonies (Figures 3B and 5). Smaller fitness effects can be observed with comparable sensitivity by increasing the number of replicates per strain (Supplementary Figure S14A). Existing methods, like MCAT (Bean *et al.* 2014), also provide quantitative output of fitness with high resolution; however, without a reference grid and proper spatial bias correction, one cannot statistically determine if the small effects are meaningful. LI Detector’s ability to detect small increases in fitness, in particular, makes it a favorable method to examine gain-of-function mutations, questions of evolutionary biology, and pharmacological screens of adaptation and resistance (Tong *et al.* 2001; Parsons *et al.* 2004; Nichols *et al.* 2011; Hoepfner *et al.* 2014; Lee *et al.* 2014; Piotrowski *et al.* 2017; Durand *et al.* 2019; Eberlein *et al.* 2019; Helsen *et al.* 2020; Vakirlis *et al.* 2020). The unprecedented sensitivity of LI Detector augments the discovery potential of CBHTS.

LI Detector is a flexible framework whereby the statistical strategy can be adapted to study design. For example, spatial bias correction and empirical testing for significant fitness deviations can either be performed using a single reference strain or separate reference and tester strains (Supplementary Figure S7). This enables the user, for example, to conduct spatial bias correction for a variety of different experimental conditions using a common strain while using different strains to quantitate fitness deviations in each condition, or vice-versa. At the analytical level, alternate statistical testing strategies can be incorporated without compromising sensitivity and specificity (Supplementary Figure S8C)**.**

A caveat of LI Detector is that a portion of the colony positions on the plates is sacrificed for reference colonies that could otherwise be used for mutants. Consequently, this increases the overall resources required for the experiment, including media, number of plates, storage space, pinning time, and imaging time. We have shown that LI Detector’s accuracy in predicting background colony sizes and its sensitivity in detecting small fitness effects is directly related to the proportion of reference colonies on a plate (Figure 5). However, the proportion of references per plate and the number of replicates per strain can be tunable according to the user’s requirement. It must be noted that the cost of reducing the number of references is lower for detecting more substantial fitness effects. For example, sacrificing 12.5% of the plate for reference colonies instead of 25% has almost no detriment to detecting 7% fitness effects (Figure 5, Supplementary Figure S14A). A higher number of references and replicates can be used if the goal is to look for minute changes in fitness, as are frequently observed with the deletion of nonessential genes or minor changes to the coding sequence of a given gene. Alternatively, fewer references and replicates may be used where larger fitness effects are expected or desired, such as finding the most drug-resistant mutant. That said, users interested in large fitness effects exclusively may use existing methods like MCAT (Bean *et al.* 2014) instead of the LI Detector to save resources, as long as *a priori* assumptions of the DFE are reasonable to make.

In summary, the LI Detector framework experimentally introduces a reference population grid on plates whose colony size estimates are used to correct for spatial bias independently of the underlying DFE. It has the potential to expand the utility of CBHTS by making them independent of scale, sensitive toward small fitness effects, and equally sensitive in detecting increases and decreases in fitness. LI Detector also provides a robust and reliable method to analyze 6144-format CBHTS, where a larger number of strains and replicates can be characterized simultaneously. Although developed and validated using *S. cerevisiae*, it can be applied to any colony-forming-microorganisms, including clinically relevant isolates, as long as they can be grown in the laboratory.
